# ZFN-mediated gene targeting of the Arabidopsis *protoporphyrinogen oxidase* gene through *Agrobacterium*-mediated floral dip transformation

**DOI:** 10.1111/pbi.12040

**Published:** 2012-12-28

**Authors:** Sylvia Pater, Johan E Pinas, Paul J J Hooykaas, Bert J Zaal

**Affiliations:** Department of Molecular and Developmental Genetics, Institute of Biology, Leiden UniversityLeiden, The Netherlands

**Keywords:** *Agrobacterium*-mediated floral dip transformation, Arabidopsis, gene targeting, *protoporphyrinogen oxidase*, zinc finger nucleases

## Abstract

Previously, we showed that ZFN-mediated induction of double-strand breaks (DSBs) at the intended recombination site enhanced the frequency of gene targeting (GT) at an artificial target locus using *Agrobacterium*-mediated floral dip transformation. Here, we designed zinc finger nucleases (ZFNs) for induction of DSBs in the natural *protoporphyrinogen oxidase* (*PPO*) gene, which can be conveniently utilized for GT experiments. Wild-type Arabidopsis plants and plants expressing the ZFNs were transformed via floral dip transformation with a repair T-DNA with an incomplete *PPO* gene, missing the 5′ coding region but containing two mutations rendering the enzyme insensitive to the herbicide butafenacil as well as an extra *Kpn*I site for molecular analysis of GT events. Selection on butafenacil yielded 2 GT events for the wild type with a frequency of 0.8 × 10^−3^ per transformation event and 8 GT events for the ZFNs expressing plant line with a frequency of 3.1 × 10^−3^ per transformation event. Molecular analysis using PCR and Southern blot analysis showed that 9 of the GT events were so-called true GT events, repaired via homologous recombination (HR) at the 5′ and the 3′ end of the gene. One plant line contained a *PPO* gene repaired only at the 5′ end via HR. Most plant lines contained extra randomly integrated T-DNA copies. Two plant lines did not contain extra T-DNAs, and the repaired *PPO* genes in these lines were transmitted to the next generation in a Mendelian fashion.

## Introduction

Genetic modification of plants is now routinely performed. Transformation can be performed by various methods and vectors including *Agrobacterium tumefaciens*. It has been observed that transgenes integrate at fairly random positions and in variable copy numbers in the plant genome. This variation may cause position-dependent expression or even silencing of transgenes and also mutation of endogenous genes at the integration sites. Therefore, it would be an advantage if integration could be targeted to a specific locus, in particular a gene which one wants to modify.

Targeted DNA integration via gene targeting (GT) by homologous recombination (HR) is efficient in yeast but a very rare event in somatic cells of higher eukaryotes, like animals and plants. In plants, the GT frequency varies considerably depending on the plant species. In lower plants such as the moss *Physcomitrella patens,* integration of foreign DNA predominantly occurs via HR when the incoming DNA is flanked by stretches of 50–200 bp homology to the target site (Schaefer, 2002). In higher plants, however, integration occurs by nonhomologous end joining (NHEJ), even when much longer flanking homologous sequences are used. Estimates of GT frequencies in various plant species vary from 10^−4^ to 10^−6^ (Even-Faitelson *et al*., 2011; Halfter *et al*., 1992; Hanin *et al*., 2001; Hrouda and Paszkowski, 1994; Lee *et al*., 1990; Miao and Lam, 1995; Offringa *et al*., 1990; Paszkowski *et al*., 1988; Riseeuw *et al*., 1995). In rice, however, GT frequencies appear to be relatively high, and using positive–negative selection strategies, a number of endogenous loci were successfully targeted (Iida and Terada, 2005; Terada *et al*., 2007). Such positive–negative selection procedures do not seem to work very efficiently in other plants, such as Arabidopsis (Gallego *et al*., 1999; Wang *et al*., 2001). Alternative strategies for improving GT thus had to be explored.

Gene-targeting frequencies can be increased by the introduction of a DNA double-strand break (DSB) near the site of the desired recombination event. In plants, this was demonstrated for the first time through the use of the rare cutting meganuclease I-*Sce*I, resulting in an increase in GT frequency by two orders of magnitude (Puchta *et al*., 1996). To create a DSB at a predetermined site in the genome, zinc finger nucleases (ZFNs) have been used as the tools of choice (Klug, 2010). Recently, TAL effector nucleases (TALENs) are rapidly emerging as an alternative because there seem to be fewer constraints in their DNA binding ability (Bogdanove and Voytas, 2011). The current generations of ZFNs and TALENs combine the nonspecific cleavage domain of the *Fok*I restriction enzyme with a specific DNA binding domain to provide cleavage specificity. Expression of these site-specific nucleases will induce a DSB at their recognition sites. Upon imperfect repair via NHEJ, this results in the formation of mutations, mostly small deletions. In several plant species and at various loci, this method has been successfully used (Cai *et al*., 2009; Cermak *et al*., 2011; Curtin *et al*., 2011; Lloyd *et al*., 2005; Mahfouz *et al*., 2011; Osakabe *et al*., 2010; de Pater *et al*., 2009; Shukla *et al*., 2009; Tovkach *et al*., 2009; Zhang *et al*., 2010). When a repair template is present simultaneously with the DSB induction, repair may occur via HR resulting in GT. There are only a few examples where GT events have been obtained using ZFNs for induction of DSB at the site of recombination. Most of these experiments were performed with artificial loci located on pre-inserted target sequences (de Pater *et al*., 2009; Wright *et al*., 2005). Homology-dependent gene addition at the maize inositol-1,3,4,5,6-pentakisphosphate 2-kinase gene (*IPK*) (Shukla *et al*., 2009) and GT at the tobacco acetolactate synthase gene (*ALS*) (Townsend *et al*., 2009) were achieved using ZFNs, but the effect of the ZFNs on the frequency was not assessed. In most studies, the GT repair constructs and ZFN expression constructs were codelivered to cell suspensions (Cai *et al*., 2009; Shukla *et al*., 2009) or protoplasts (Townsend *et al*., 2009; Wright *et al*., 2005) via a variety of direct DNA transformation procedures. However, for many plant species, like Arabidopsis, no efficient protoplast or cell suspension transformation and regeneration protocol are available. Therefore, we have employed the widely used bacterial vector *Agrobacterium tumefaciens* to obtain GT events in Arabidopsis (de Pater *et al*., 2009) using the floral dip transformation method. An alternative strategy, which released a pre-inserted GT repair construct from the genome by nucleases, while simultaneously producing a DSB at the site of intended recombination, was recently found to be a powerful method to obtain GT events at a high frequency in Arabidopsis (Fauser *et al*., 2012). With this method, all cells not only express the nuclease but also contain the GT repair construct.

Several years ago, a very efficient selection system for GT at the *protoporphyrinogen oxidase* (*PPO*) gene has been developed (Hanin *et al*., 2001). The *PPO* gene is involved in chlorophyll and haem synthesis. The encoded enzyme can be inhibited by the herbicide butafenacil, which leads to rapid plant death due to formation of reactive oxygen. Two specific amino acid changes render this enzyme insensitive for the herbicide, which can be exploited for selection of GT events by the introduction of these mutations in the *PPO* gene via HR.

As we demonstrated previously for an artificial target locus (de Pater *et al*., 2009), Arabidopsis plants expressing ZFNs showed increased GT frequencies via the widely used floral dip transformation method with *Agrobacterium tumefaciens*. Here, we report GT experiments at the natural *PPO* locus with plants expressing ZFNs for DSB induction at this locus, compared to plants without ZFNs. It was shown that the frequency of precise GT events at this locus is enhanced by the expression of ZFNs.

## Results and Discussion

### Design, cloning and expression of ZFNs for DSB induction in the PPO gene

Previously, we showed that induction of DSBs using ZFNs enhanced the GT frequency at an artificial locus (de Pater *et al*., 2009). The strategy that was used included three consecutive transformation steps. First, the artificial target site was introduced, then the ZFNs and finally the GT repair construct, using the commonly used *Agrobacterium*-mediated floral dip transformation. This strategy made it possible to check whether the ZFN genes were present and expressed before the final GT experiment. In this manner, cells transformed with the GT repair construct already contain the ZFNs, thus avoiding the necessity of introducing all required constructs at once. Although theoretically possible, cotransformation with multiple T-DNAs delivered by a mixture of *Agrobacterium* strains will only result in very few transformants containing all components. Although the ZFNs were expressed over a long period of time, only a small fraction of the target sites in these plants contained footprints. Apparently, the majority of the induced DSBs were repaired precisely.

**Figure 1:**
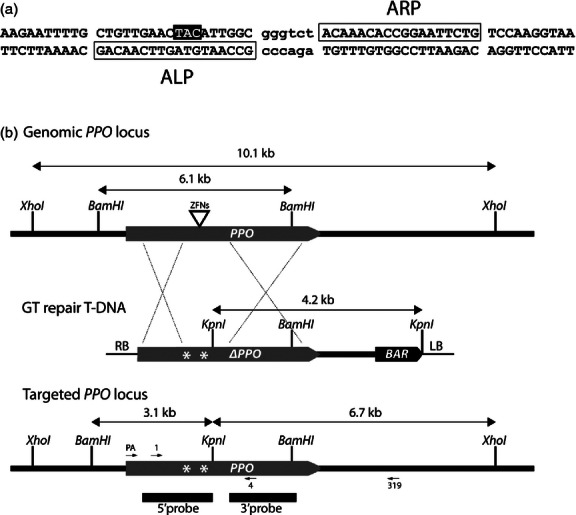
Structures of the wild-type genomic *PPO* locus, the T-DNA repair gene-targeting (GT) construct and the targeted *PPO* locus. (a) The sequence of the zinc finger nucleases (ZFN) target site is shown, the target half sites for ALP and ARP are boxed and the spacer is in small letters. The triplet coding for tyrosine in the endogenous *PPO* gene (and changed in the repair construct) is shown in a black box. (b) The coding region of the *PPO* gene is shown as a grey bar; the ZFN target site in the *PPO* gene as a triangle. The GT repair construct, missing the C-terminal region of the *PPO* gene including the first 364 bps of the coding sequence, contains base pair substitutions leading to two amino acids changes (S305L and Y426M; indicated by asterisks) causing insensitivity for the herbicide butafenacil and a *Kpn*I site for detection of GT events. The *BAR* gene on the GT repair construct is used to determine the transformation frequency. Primers used for PCR detection of GT events are shown. Sizes of DNA fragments expected after digestion with selected restriction enzymes are indicated. Probes used for Southern blotting are shown as black bars.

To enhance the GT frequency at the natural *PPO* locus, a similar strategy was followed, except for the introduction of the target site, which was already present in the natural situation. ZFNs for generating DSBs at the *PPO* locus were designed. Based upon our previous work (de Pater *et al*., 2009), we wanted to use 6-fingered (6F) ZF domains for target site recognition, as these larger DNA recognition domains yielded the most active ZFNs in our assembly system. To avoid ZFN-mediated digestion of the incoming GT repair construct, we aimed to have one of the ZF domains more specifically recognizing the endogenous *PPO* sequence compared to the incoming *PPO* sequence present on the T-DNA. This could be achieved by incorporating a ZF designed to recognize the 3-bp sequence TAC, encoding tyrosine in the wild-type *PPO* sequence, rather than for ATG, encoding methionine in the repair construct, which is one of the two amino acid changes conferring butafenacil resistance. Having this constraint for target site selection was another reason for aiming at the longer 6F ZF domains as these should still allow for adequate 2 × 18 bp target site recognition, despite that some suboptimal ZF–DNA interactions had to be included. It was convenient to use the online Zinc Finger Tools software (http://www.zincfingertools.org) for target site and ZF selection. The final ZFN target site (CTG TTG AAC TAC ATT GGC gggtct ACA AAC ACC GGA ATT CTG) was chosen as meeting our demands. The ZF domain recognizing the left half of the target site was designated ALP, and the ZF domain recognizing the right half of the target site was designated ARP (Figure 1a). These ZF domains were linked to the FOK nuclease domains encoded by T-DNA vectors, resulting in *ALPFOK* and *ARPFOK* (Figure S1).

Arabidopsis plants were transformed through *Agrobacterium*-mediated floral dip transformation with a mixture of two *Agrobacterium* strains containing either *ALPFOK* or *ARPFOK* under control of the CaMV 35S promoter. Transformants were selected on kanamycin and hygromycin for the presence of both T-DNAs. PCR analysis showed that in several plant lines, both ZFN genes were present in equimolar amounts (Figure S2A). Two lines were chosen for further analysis by RT-qPCR. At the mRNA level, both ZFN-encoding genes in these lines were expressed at the same order of magnitude as the 35S-ZFN genes in plants produced previously (de Pater *et al*., 2009) (Figure S2B). As the phenotype of both selected plant lines appeared to be normal, the presence of the ZFN pairs in these plants did not cause any toxic effects. PCR amplification and sequencing of the target site showed that the majority was unchanged, like we found before with the artificial target site (de Pater *et al*., 2009). Plant line number 3 (hereafter indicated as line T) was chosen for transformation with the GT repair construct (Figure 1b). As a control, wild-type plants were used for comparison of the GT frequency in the absence of ZFNs.

### Screening for GT events at the PPO locus

To determine the GT frequency (number of GT events per (random) integration event), the transformation frequency (random integration) was first determined. Aliquots of the seeds obtained after floral dip transformations were selected for the presence of the *BAR* gene at the 3′ end of the T-DNA on phosphinothricin containing medium. The presence of the *BAR* gene reflects those transformation events where a complete T-DNA was inserted at a random position in the genome. For the wild type, 1.3% of the seeds was transformed and for the T line containing the ZFNs 1.6%. Subsequently, 2 × 10^5^ seeds derived from the wild type and 1.6 × 10^5^ seeds derived from the T line were selected on butafenacil for identification of GT events, both seed samples corresponding to 2600 transformants. Putative butafenacil-resistant plants were allowed to set seed, which was selected again on butafenacil. Two butafenacil-resistant plant lines (C1 and C2) were obtained for the wild type and eight plant lines for the T line (T1-T8). Subsequently, these plant lines were also tested on phosphinothricin. Plant lines C1 and T2 were sensitive to phosphinothricin, whereas the other lines were phosphinothricin resistant, indicating that they probably contain extra randomly integrated T-DNAs.

### Molecular analysis of GT events

**Figure 2:**
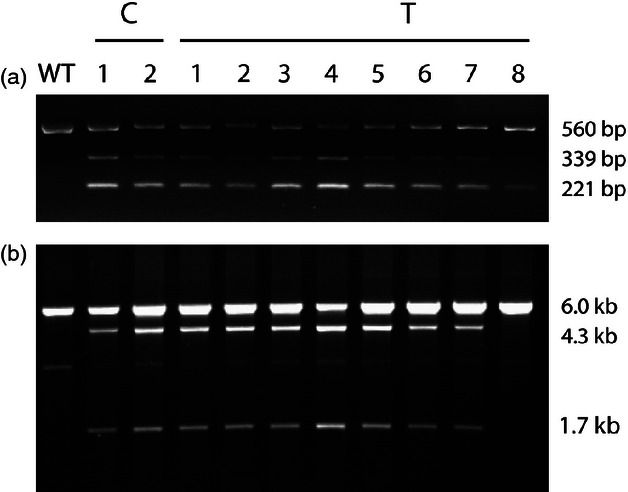
PCR analysis of butafenacil-resistant gene-targeting (GT) plants. PCR was performed on wild-type (WT), 2 butafenacil-resistant plant lines not transformed with zinc finger nucleases (ZFNs) constructs (C1 and C2) and 8 butafenacil-resistant lines expressing the ALP and ARP ZFNs (T1-T8). Primers PPO-PA and PPO-4 were used, followed by nested PCR with primers PPO-1 and PPO-4 (a), or primers PPO-PA and SP319 were used (b). PCR products were digested with *Kpn*I and analysed on 1.5% (a) or 0.7% (b) agarose gels. The sizes of the bands are shown.

The butafenacil-resistant plants were analysed by PCR. First, the 5′ recombination region was amplified with primers PPO-PA and PPO-4 followed by nested PCR with primers PPO-1 and PPO-4 (Figure 1b). The products (560 bp) were digested with *Kpn*I for identification of GT events. All butafenacil-resistant plants produced *Kpn*I-sensitive PCR products, resulting in bands of 339 bp and 221 bp (Figure 2a), indicating GT via HR between the genomic *PPO* locus and the GT repair construct on the T-DNA at the 5′ end of the gene. All plants also contained PCR products that were resistant to *Kpn*I indicating that the wild-type locus was still present and that the plants were heterozygous for the *PPO* locus. To analyse whether integration at the 3′ end of the gene also took place via HR, PCR was performed with primers PPO-PA and SP319, both located on the genomic locus, but absent in the GT construct. The PCR products of 6.0 kb were digested with *Kpn*I. For all plants except T8, *Kpn*I-sensitive products were obtained, resulting in bands of 4.3 and 1.7 kb (Figure 2b), indicating that in 9 of the 10 GT plant lines, true GT (TGT) had occurred.

**Figure 3:**
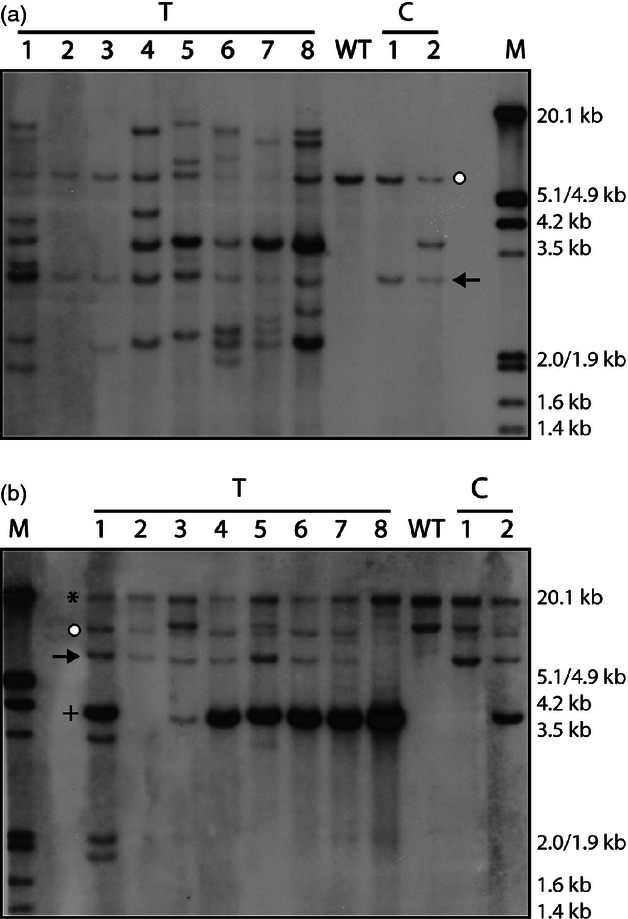
Southern blot analysis of gene-targeting (GT) plant lines. Ten μg DNA of wild-type plants (WT) or butafenacil-resistant plants (C and T) was digested with *Kpn*I and *Bam*HI (a) or *Kpn*I and *Xho*I (b), separated on 0.7% agarose gels and hybridized with a 5′*PPO* probe (a) or 3′*PPO* probe (b). The dots indicate wild-type bands (6.7 and 10.1 kb). Arrows indicate bands with the expected sizes (3.1 and 6.1 kb) after GT by homologous recombination (HR). The plus represents the internal 4.2 kb *Kpn*I fragment present on randomly integrated T-DNAs. The asterisk indicates approximately 20-kb bands probably resulting from partial digestion. Lanes M contain DIG-labelled lambda *Eco*RI/*Hind*III marker. The sizes of the marker bands are shown.

To exclude that the *Kpn*I-sensitive PCR products were artefacts resulting from the PCR, Southern blot analysis was performed. DNA was digested with *Kpn*I and *Bam*HI for hybridization with the 5′ probe (Figure 3a) or digested with *Kpn*I and *Xho*I for hybridization with the 3′ probe (Figure 3b). The expected sizes of the 5′ and 3′ GT bands are 3.1 kb and 6.7 kb, respectively (Figure 1b). The expected bands were present in all plant lines except for the 3′ GT band in line T8. As mentioned above, this line also did not produce a *Kpn*I-sensitive PCR product with primers PPO-PA and SP319 and represented therefore not a TGT event. These results thus proved that indeed, 9 of 10 GT lines represented perfect TGT events. This proportion is much higher than previously reported in GT experiments with the PPO gene where about one-third of the events were TGT (Hanin *et al*., 2001; Jia *et al*., 2012) and is possibly the result of the use of ZFNs for DSB induction.

The GT frequency after ZFN-mediated DSB induction was 3.1 × 10^−3^ per transformation event (TGT frequency of 2.7 × 10^−3^). The GT frequency of the wild-type plants was 4 times lower (0.8×10^−3^) and was comparable to that previously found by others (Hanin *et al*., 2001). In current models for HR pathways, one of the key features is that HR is initiated by a DSB (San Filippo *et al*., 2008). Therefore, it is most likely that the GT events in the wild type probably resulted via DSBs that had occurred spontaneously.

In most lines, many extra bands were present after hybridization with the 5′ probe, indicating that extra T-DNAs had been randomly integrated (Figure 3a). In lines C1 and T2, only the wild-type and GT bands were visible, indicating that these lines did not contain extra randomly integrated T-DNAs. In most lanes, hybridization with the 3′ probe resulted in a thick band representing the internal 4.2 kb *Kpn*I fragment of the randomly integrated T-DNA copies (Figures 1b and 3b).

**Figure 4:**
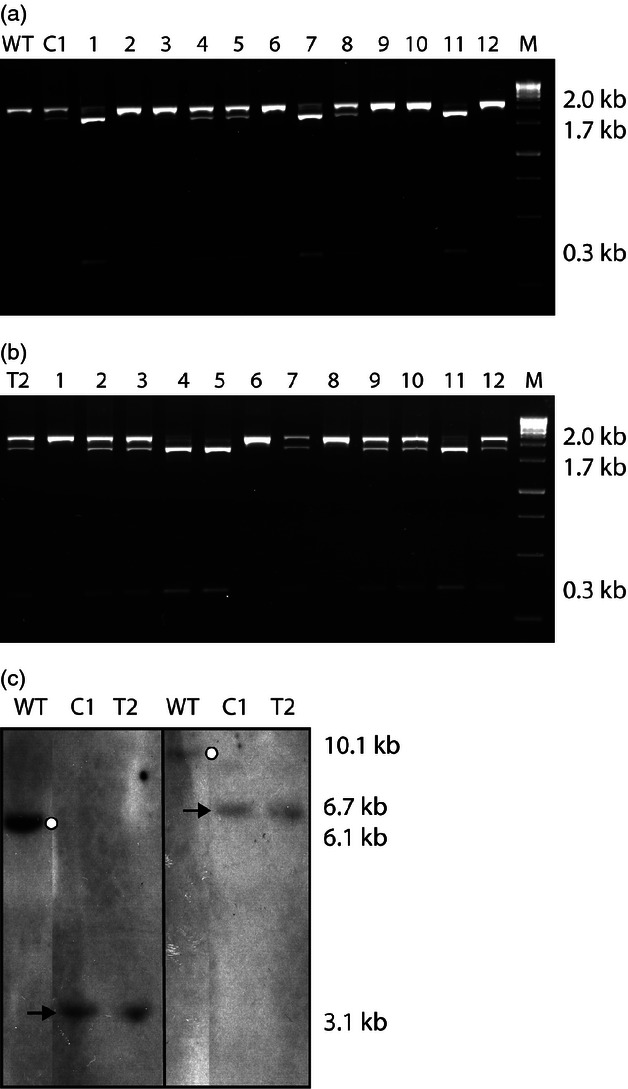
Analysis of progeny gene-targeting (GT) plants. PCR analysis was performed on wild-type (WT), the C1 GT plant and 12 C1 progeny plants (a) and the T2 GT plant and 12 T2 progeny plants (b), using primers PA and PPO-4, followed by *Kpn*I digestion of the 2.0-kb PCR products. C1 progeny plants 2, 3, 6, 9, 10 and 12 were wild type (2.0-kb band), C1 progeny plants 1, 7 and 11 were homozygous GT (1.7- and 0.3-kb bands), and C1 progeny plants 4, 5 and 8 were heterozygous (2.0-, 1.7- and 0.3-kb bands). T2 progeny plants 1, 6 and 8 were wild type (2.0-kb band), T2 progeny plants 4, 5 and 11 were homozygous GT (1.7- and 0.3-kb bands), and T2 progeny plants 2, 3, 7, 9, 10 and 12 were heterozygous (2.0-, 1.7- and 0.3-kb bands). The DNA fragments were separated on 1.5% agarose gels. Lane M contains 1-kb marker (Fermentas). Southern blot analysis (c) with DNA from wild-type and homozygous C1 and T2 progeny plants digested with *Kpn*I and *Bam*HI (left panel) or *Kpn*I and *Xho*I (right panel), separated on 0.7% agarose gels and hybridized with a 5′*PPO* probe (left panel) or 3′*PPO* probe (right panel). Arrows indicate bands with the expected sizes (3.1 and 6.7 kb) after GT by homologous recombination (HR). The dots indicate bands with the expected sizes (6.1 and 10.1 kb) of wild-type bands.

Subsequently, progeny plants were analysed for the segregation of the wild-type and GT *PPO* locus. To simplify the analysis, the C1 and T2 GT lines were chosen, because they do not contain additional T-DNA copies. PCR with primers PPO-PA and PPO-4 resulted in 2.0-kb products. *Kpn*I digestion showed that the bands in the C1 progeny plants 2, 3, 6, 9, 10 and 11 and the T2 progeny plants 1, 6 and 8 were insensitive to *KpnI* (2.0 kb), indicating that in these plants, the wild-type locus was present. The bands in the C1 progeny plants 1, 7 and 11 and the T2 progeny plants 4, 5 and 11 were sensitive to *KpnI*, resulting in bands of 1.7 kb and 0.3 kb, indicating that they were homozygous for the GT locus. The remaining C1 and T2 progeny plants contained three bands of 2.0, 1.7 and 0.3 kb, indicating that these were heterozygous for the GT locus. These results showed that Mendelian segregation occurred of the two alleles for both plant lines (Figure 4a). Homozygous C1 and T2 progeny plant lines were also used for Southern blot analysis. As shown in Figure 4b, these lines contained only the expected 5′ and 3′ GT bands of 3.1 and 6.7 kb, respectively.

These results together showed that TGT plants were obtained and that the repaired *PPO* loci were transmitted to the next generation as expected. The two-step method with *Agrobacterium*-mediated floral dip transformations that we followed is very useful for GT in *Arabidopsis*, because it is very easy and does not rely on complicated transformation and regeneration protocols. The increase in GT events that we observed for the endogenous *PPO* gene using ZFNs is rather promising for the ability to precisely modify any natural target sites at high frequency.

## Experimental procedures

### Construction of the GT repair construct, ZFN expression vectors and plant transformation

The *PPO* GT construct was created by overlap PCR on Arabidopsis ecotype Col-0 DNA as a template using primers to create two mutations for butafenacil resistance (S305L and Y426M) and a *Kpn*I site at position E445/A446 for identification of GT events. The obtained *PPO* gene missing the first 364 bp of the coding sequence and containing 3660 bp of the 3′ noncoding sequence was cloned in pCambia3200 (digested with *Xma*I/*PmeI*), creating pSDM3900.

Sequences encoding polydactyl zinc fingers (PZFs), targeted to the *PPO* gene, were assembled according to the method previously described (Neuteboom *et al*., 2006). Oligonucleotides coding for ZF domains that bind particular triplets for cloning the ZF domains are shown in Table S1. The target sequence for the ZFN pair is *CTG TTG AAC* *TAC* *ATT GGC* gggtct ACA AAC ACC GGA ATT CTG (italic: bottom strand is recognized by ZFN; underlined: mutation Y426M; small font: spacer) (Figure 1a). The 6F ZF domains (named ALP and ARP) were exchanged with the ZF domains present in pSDM3838 (kanamycin selection) and pSDM3839 (hygromycin selection) (de Pater *et al*., 2009), resulting in pSDM3901 and pSDM3902, respectively. The ZFNs in these plasmids are driven by the 35S promoter. The accession numbers of *ALPFOK* and *ARPFOK* are BankIt1581546 KC164376 and BankIt1581546 KC164377, respectively, and the nucleotide and amino acid sequences are shown in Figure S2.

Plant vectors were introduced in *Agrobacterium tumefaciens* AGL1 (Lazo *et al*., 1991) by electroporation. Arabidopsis plants (ecotype Col-0) were transformed via the floral dip method (Clough and Bent, 1998), and primary transformants were selected on MA solid medium without sucrose supplemented with nystatin (100 μg/mL), timentin (100 μg/mL) and the appropriated antibiotics or herbicide: 15 μg/mL hygromycin and 30 μg/mL kanamycin for selection of plants transformed with the ZFNs; 15 μg/mL phosphinothricin for transformation frequency of the GT repair construct carrying the *BAR* gene; 50 μM butafenacil for selection of GT events.

### DNA isolation and PCR analysis

Seedlings, leaves or flowers were disrupted to a powder under liquid N_2_ in a TissueLyser (Retch, Haan, Germany). DNA was isolated as described (de Pater *et al*., 2009). One μl (usually 0.1 μg DNA) was used for PCR in a final volume of 25 μL with either REDTaq™ (Sigma-Aldrich, St Louis, MO) or Phusion (Finnzymes, Espoo, Finland) polymerase. PCR primers are shown in Table S2. For amplification of ZFN coding regions, primers SP258 and SP259 were used. For detection of GT events, the primers PPO-PA, PPO-1, PPO-4 and SP319 were used in various combinations, followed by digestion of the PCR products with *Kpn*I.

### RNA isolation and RT-q-PCR

For expression analysis of ZFN constructs, pools of 10-day-old seedlings were frozen in liquid N_2_. RNA isolation and RT-PCR were performed as described (de Pater *et al*., 2009). Expression of ZFNs was analysed with primers SP258 and SP286 for the *ALP* ZF domain, with primers SP258 and SP281 for the *ARP* ZF domain, with primers SP258 and SP274 for the E2C ZF domain, with primers SP258 and SP273 for the PTF ZF domain and with primers SP272 and SP259 for the *FOK* domain. Normalization of relative gene expression was based on expression of the housekeeping gene *ROC1* (primers ROC3.3, ROC5.2).

### Southern blot analysis

Plant DNA (10 μg) was digested with *Kpn*I and *Bam*HI (for analysis of the 5′ recombination site) or *Kpn*I and *Xho*I (for analysis of the 3′ recombination site) and separated in 0.7% agarose gels, blotted onto Hybond-N and hybridized in DIG Easy Hyb (Roche Diagnostics, Mannheim, Germany) supplemented with 50 μg/mL herring sperm DNA with a 5′ *PPO* probe (primers SP154 and SP155) or a 3′ *PPO* probe (primers SP156 and SP157), labelled in a PCR using DIG-labelling mix (Roche Diagnostics, Mannheim, Germany). After 16–20 h, blots were washed twice with 2×SSC; 0.1% SDS at room temperature and three times with 0.2×SSC; and 0.1% SDS at 65 °C. Detection was performed using the DIG wash and block buffer set and CDP-Star (Roche Diagnostics, Mannheim, Germany) according the manufacturers protocol.

## Supporting information

**Figure S1** Nucleotide and amino acid sequence of *ALPFOK* and *ARPFOK*.

**Figure S2** Analysis of ZFN transformed Arabidopsis lines.

**Table S1** Oligonucleotides for cloning ZF domains.

**Table S2** Primers used for PCR reactions.
