# Supplementary material for: ZFN-mediated gene targeting of the Arabidopsis protoporphyrinogen oxidase gene through Agrobacterium-mediated floral dip transformation
Source: Plant Biotechnol J. 2012 Dec 28;11(4):510–5. doi: 10.1111/pbi.12040 (PMC3719044; doi:10.1111/pbi.12040)
Supplement: Supplementary file 1 [file pbi0011-0510-SD1.pdf]

ALPFOK

Nucleotide

ATGGCCGACTACAAGGACGACGACGATAAGCGGCCGCTTGAGCCTCCGAAAAAGAAGCGTAAG
GTCGAGCTAGCAGGTACCGGGATCCTGCAGGATATCGATCTCGAGGCCCAGGCGGCCCTCGAA
CCCACCGGCGAGAAGCCCTACGCTTGCCCGGAGTGTGGCAAGTCATTCTCCAGGGCTGACAAT
TTGACTGAACATCAACGCACACATACC
GGCGAGAAACCGTACGCTTGCCCTGAATGCGGAAAAAGCTTCTCTCAGTCCGGTAACCTCACA
GAACATCAGAGGACCCATACCGGCGAGAAACCCTACGCCTGCCCGGAATGCGGGAAGTCGTTT
AGCACCTCCGGTAGCCTGGTGAGGCATCAGCGTACGCATACCGGCGAGAAGCCTTACGCTTGC
CCCGAGTGTGGCAAATCGTTCTCCCAGTCCTCCTCGTTGGTCCGTCATCAACGGACGCATACC
GGCGAGAAGCCTTATGCCTGTCCTGAGTGCGGGAAGTCTTTTAGCACCACAGGTAATCTCACT
GTCCACCAACGTACGCATACCGGCGAGAAGCCTTACGCTTGCCCGGAGTGTGGCAAGTCATTC
AGCGACTGCCGTGATCTCGCAAGGCATCAACGCACCCATACC
GGCGAGCTGGGTGGCGGTTCCGGAGAAAAGCCCGGGAAGAAAACCTCGGGCCAGGCCGGC
CAACTAGTCAAAAGTGAACTGGAGGAGAAGAAATCTGAACTTCGTCATAAATTGAAATATGTG
CCTCATGAATATATTGAATTAATTGAAATTGCCAGAAATTCCACTCAGGATAGAATTCTTGAA
ATGAAGGTAATGGAATTTTTTATGAAAGTTTATGGATATAGAGGTAAACATTTGGGTGGATCA
AGGAAACCGGACGGAGCAATTTATACTGTCGGATCTCCTATTGATTACGGTGTGATCGTGGAT
ACTAAAGCTTATAGCGGAGGTTATAATCTGCCAATTGGCCAAGCAGATGAAATGCAACGATAT
GTCGAAGAAAATCAAACACGAAACAAACATATCAACCCTAATGAATGGTGGAAAGTCTATCCA
TCTTCTGTAACGGAATTTAAGTTTTTATTTGTGAGTGGTCACTTTAAAGGAAACTACAAAGCT
CAGCTTACACGATTAAATCATATCACTAATTGTAATGGAGCTGTTCTTAGTGTAGAAGAGCTT
TTAATTGGTGGAGAAATGATTAAAGCCGGCACATTAACCTTAGAGGAAGTGAGACGGAAATTT
AATAACGGCGAGATAAACTTTTAA

Protein

MADYKDDDDKRPLEPPKKKRKVELAGTGILQDIDLEAQAALEPT
GEKPYACPECGKSFSRADNLTEHQRTHT
GEKPYACPECGKSFSQSGNLTEHQRTHT
GEKPYACPECGKSFSTSGSLVRHQRTHT
GEKPYACPECGKSFSQSSSLVRHQRTHT
GEKPYACPECGKSFSTTGNLTVHQRTHT
GEKPYACPECGKSFSDCRDLARHQRTHT
GELGGGSGEKPGKKTSGQAG
QLVKSELEEKKSELRHKLKYVPHEYIELIEIARNSTQDRILEMKVMEFFMKVYGYRGKHLGGS
RKPDGAIYTVGSPIDYGVIVDTKAYSGGYNLPIGQADEMQRYVEENQTRNKHINPNEWWKVYP
SSVTEFKFLFVSGHFKGNYKAQLTRLNHITNCNGAVLSVEELLIGGEMIKAGTLTLEEVRRKF
NNGEINF

ARPFOK

Nucleotide

ATGGCCGACTACAAGGACGACGACGATAAGCGGCCGCTTGAGCCTCCGAAAAAGAAGCGTAAG
GTCGAGCTAGCAGGTACCGGGATCCTGCAGGATATCGATCTCGAGGCCCAGGCGGCCCTCGAA
CCCACCGGCGAAAAGCCGTATGCGTGCCCCGAATGTGGGAAGTCCTTTTCTCGTAATGACGCT
CTTACAGAACACCAAAGGACCCATACC
**GGCGAGAAACCCTACGCCTGCCCGGAATGCGGGAAGTCGTTTAGCCATAAGAACGCTCTGCAG
AATCATCAGCGTACGCATACCGGCGAGAAACCATACGCCTGTCCCGAGTGCGGTAAAAGCTTT
AGTCAGCGTGCCCATCTTGAACGCCACCAGAGGACCCATACCGGCGAGAAGCCTTACGCTTGC
CCGGAGTGTGGCAAGTCATTCAGCGACAAAAAGGATCTCACTAGGCATCAACGCACCCATACC
GGCGAGAAGCCCTACGCATGCCCGGAATGCGGAAAATCCTTCTCCGATTCCGGTAACTTGCGT
GTTCATCAGCGCACCCATACCGGCGAGAAGCCCTACGCCTGCCCGGAGTGTGGAAAGTCGTTC
TCCTCACCTGCAGATTTGACACGGCATCAACGGACGCATACC**
GGCGAGCTGGGTGGCGGTTCCGGAGAAAAGCCCGGGAAGAAAACCTCGGGCCAGGCCGGC
CAACTAGTCAAAAGTGAACTGGAGGAGAAGAAATCTGAACTTCGTCATAAATTGAAATATGTG
CCTCATGAATATATTGAATTAATTGAAATTGCCAGAAATTCCACTCAGGATAGAATTCTTGAA
ATGAAGGTAATGGAATTTTTTATGAAAGTTTATGGATATAGAGGTAAACATTTGGGTGGATCA
AGGAAACCGGACGGAGCAATTTATACTGTCGGATCTCCTATTGATTACGGTGTGATCGTGGAT
ACTAAAGCTTATAGCGGAGGTTATAATCTGCCAATTGGCCAAGCAGATGAAATGCAACGATAT
GTCGAAGAAAATCAAACACGAAACAAACATATCAACCCTAATGAATGGTGGAAAGTCTATCCA
TCTTCTGTAACGGAATTTAAGTTTTTATTTGTGAGTGGTCACTTTAAAGGAAACTACAAAGCT
CAGCTTACACGATTAAATCATATCACTAATTGTAATGGAGCTGTTCTTAGTGTAGAAGAGCTT
TTAATTGGTGGAGAAATGATTAAAGCCGGCACATTAACCTTAGAGGAAGTGAGACGGAAATTT
AATAACGGCGAGATAAACTTTTAA

Protein

MADYKDDDDKRPLEPPKKKRKVELAGTGILQDIDLEAQAALEPT
**GEKPYACPECGKSFSRNDALTEHQRTHT
GEKPYACPECGKSFSHKNALQNHQRTHT
GEKPYACPECGKSFSQRAHLERHQRTHT
GEKPYACPECGKSFSDKKDLTRHQRTHT
GEKPYACPECGKSFSDSGNLRVHQRTHT
GEKPYACPECGKSFSSPADLTRHQRTHT**
GELGGGSGEKPGKKTSGQAG
QLVKSELEEKKSELRHKLKYVPHEYIELIEIARNSTQDRILEMKVMEFFMKVYGYRGKHLGGS
RKPDGAIYTVGSPIDYGVIVDTKAYSGGYNLPIGQADEMQRYVEENQTRNKHINPNEWWKVYP
SSVTEFKFLFVSGHFKGNYKAQLTRLNHITNCNGAVLSVEELLIGGEMIKAGTLTLEEVRRKF
NNGEINF

Figure S1

Nucleotide and protein sequences of *ALPFOK* and *ARPFOK*.

The FLAG tag (DYKDDDDK) and Nuclear Localization Sequence (KKKRK) are underlined, ZF domains are shown in bold and the linker in italic. The accession numbers of *ALPFOK* and *ARPFOK* are BankIt1581546 KC164376 and BankIt1581546 KC164377, respectively.
