# Supplementary material for: ZFN-mediated gene targeting of the Arabidopsis protoporphyrinogen oxidase gene through Agrobacterium-mediated floral dip transformation
Source: Plant Biotechnol J. 2012 Dec 28;11(4):510–5. doi: 10.1111/pbi.12040 (PMC3719044; doi:10.1111/pbi.12040)
Supplement: Supplementary file 2 [file pbi0011-0510-SD2.pdf]

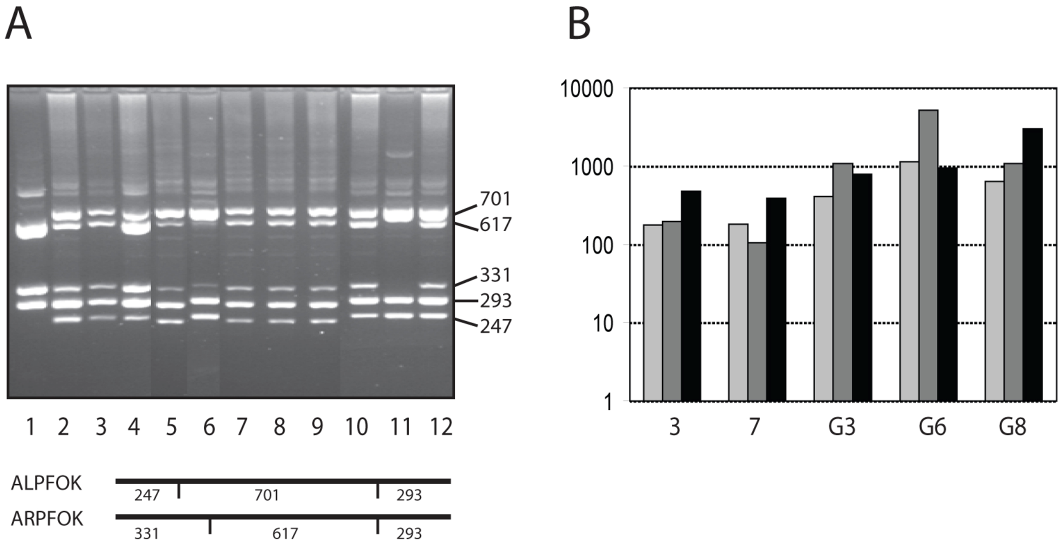

Figure S2. Analysis of ZFN transformed Arabidopsis lines.

A. Arabidopsis lines (1-12) transformed with *ALPFOK* and *ARPFOK* under control of the 35S promoter were analyzed by PCR, using primers SP258 and SP259, amplifying the complete coding region of both *ALPFOK* and *ARPFOK*, and subsequent *Hin*dIII (H) restriction analysis for the presence of both ZFNs. The expected lengths of DNA fragments obtained after amplification and digestion of ALPFOK and ARPFOK are shown below. Line 1 contained *ARPFOK*, line 11 contained *ALPFOK* and all other lines contained both genes, although not all lines showed equimolar amounts.

B. Relative expression levels of the ZFNs. Two plant lines containing equal amounts of *ALPFOK* and *ARPFOK* (3 and 7) and three plant lines containing *E2CFOK* and *PTFFOK* (G3, G6, G8; (de Pater et al. 2009)) , all under control of the 35S promoter, were analyzed by RT-QPCR using the housekeeping gene *ROC1* as control. Primer sets were used for amplification of one ZF domain (*ALP* or *E2C*; light grey), the other ZF domain (*ARP* or *PTF*; dark grey) or the *FOK* nuclease domain (black).
