# Supplementary material for: ZFN-mediated gene targeting of the Arabidopsis protoporphyrinogen oxidase gene through Agrobacterium-mediated floral dip transformation
Source: Plant Biotechnol J. 2012 Dec 28;11(4):510–5. doi: 10.1111/pbi.12040 (PMC3719044; doi:10.1111/pbi.12040)
Supplement: Supplementary file 3 [file pbi0011-0510-SD3.doc]

Supporting information

Table S1. Oligonucleotides for cloning ZF domains

| Primer | Sequence |
| --- | --- |
| GCC sense | CCGGCGAGAAGCCTTACGCTTGCCCGGAGTGTGGCAAGTCATTCAGCGACTGCCGTGATCTCGCAAGGCATCAACGCACCCATA |
| GCC antisense | CCGGTATGGGTGCGTTGATGCCTTGCGAGATCACGGCAGTCGCTGAATGACTTGCCACACTCCGGGCAAGCGTAAGGCTTCTCG |
| AAT sense | ccggcgagaagccttatgcctgtcctgagtgcgggaagtcttttagcaccacaggtaatctcactgtccaccaacgtacgcata |
| AAT antisense | ccggtatgcgtacgttggtggacagtgagattacctgtggtgctaaaagacttcccgcactcaggacaggcataaggcttctcg |
| GTA sense | CCGGCGAGAAGCCTTACGCTTGCCCCGAGTGTGGCAAATCGTTCTCCCAGTCCTCCTCGTTGGTCCGTCATCAACGGACGCATA |
| GTA antisense | CCGGTATGCGTCCGTTGATGACGGACCAACGAGGAGGACTGGGAGAACGATTTGCCACACTCGGGGCAAGCGTAAGGCTTCTCG |
| GTT sense | CCGGCGAGAAACCCTACGCCTGCCCGGAATGCGGGAAGTCGTTTAGCACCTCCGGTAGCCTGGTGAGGCATCAGCGTACGCATA |
| GTT antisense | CCGGTATGCGTACGCTGATGCCTCACCAGGCTACCGGAGGTGCTAAACGACTTCCCGCATTCCGGGCAGGCGTAGGGTTTCTCG |
| CAA sense | ccggcgagaaaccgtacgcttgccctgaatgcggaaaaagcttctctcagtccggtaacctcacagaacatcagaggacccata |
| CAA antisense | ccggtatgggtcctctgatgttctgtgaggttaccggactgagagaagctttttccgcattcagggcaagcgtacggtttctcg |
| CAG sense | ccggcgagaagccctacgcttgcccggagtgtggcaagtcattctccagggctgacaatttgactgaacatcaacgcacacata |
| CAG antisense | ccggtatgtgtgcgttgatgttcagtcaaattgtcagccctggagaatgacttgccacactccgggcaagcgtagggcttctcg |
| ACA sense | ccggcgagaagccctacgcctgcccggagtgtggaaagtcgttctcctcacctgcagatttgacacggcatcaacggacgcata |
| ACA antisense | ccggtatgcgtccgttgatgccgtgtcaaatctgcaggtgaggagaacgactttccacactccgggcaggcgtagggcttctcg |
| AAC sense | ccggcgagaagccctacgcatgcccggaatgcggaaaatccttctccgattccggtaacttgcgtgttcatcagcgcacccata |
| AAC antisense | ccggtatgggtgcgctgatgaacacgcaagttaccggaatcggagaaggattttccgcattccgggcatgcgtagggcttctcg |
| ACC sense | ccggcgagaagccttacgcttgcccggagtgtggcaagtcattcagcgacaaaaaggatctcactaggcatcaacgcacccata |
| ACC antisense | ccggtatgggtgcgttgatgcctagtgagatcctttttgtcgctgaatgacttgccacactccgggcaagcgtaaggcttctcg |
| GGA sense | CCGGCGAGAAACCATACGCCTGTCCCGAGTGCGGTAAAAGCTTTAGTCAGCGTGCCCATCTTGAACGCCACCAGAGGACCCATA |
| GGA antisense | CCGGTATGGGTCCTCTGGTGGCGTTCAAGATGGGCACGCTGACTAAAGCTTTTACCGCACTCGGGACAGGCGTATGGTTTCTCG |
| ATT sense | ccggcgagaaaccctacgcctgcccggaatgcgggaagtcgtttagccataagaacgctctgcagaatcatcagcgtacgcata |
| ATT antisense | ccggtatgcgtacgctgatgattctgcagagcgttcttatggctaaacgacttcccgcattccgggcaggcgtagggtttctcg |
| CTG sense | ccggcgaaaagccgtatgcgtgccccgaatgtgggaagtccttttctcgtaatgacgctcttacagaacaccaaaggacccata |
| CTG antisense | ccggtatgggtcctttggtgttctgtaagagcgtcattacgagaaaaggacttcccacattcggggcacgcatacggcttttcg |
