# Supplementary material for: ZFN-mediated gene targeting of the Arabidopsis protoporphyrinogen oxidase gene through Agrobacterium-mediated floral dip transformation
Source: Plant Biotechnol J. 2012 Dec 28;11(4):510–5. doi: 10.1111/pbi.12040 (PMC3719044; doi:10.1111/pbi.12040)
Supplement: Supplementary file 4 [file pbi0011-0510-SD4.doc]

Table S2. Primers used for PCR reactions.

| Primer | Sequence | Used for |
| --- | --- | --- |
| PPO-PA | GTGACCGAGGCTAAGGATCGT | GT detection |
| PPO-1 | GCAAGGAGTTGAAACATTAG | GT detection |
| PPO-4 | CATGAAGTTGTTGACCTCAATC | GT detection |
| SP319 | CTATCAAAGAGCACAGACAGC | GT detection |
| SP258 | CGACTACAAGGACGACGACG | ZFN DNA analysis and expression |
| SP259 | CCTCTAAGGTTAATGTGCC | ZFN DNA analysis and expression |
| SP281 | CTTTGGTGTTCTGTAAGAG | ZFN expression |
| SP286 | TTGTCAGCCCTGGAGAATG | ZFN expression |
| SP272 | CCCTAATGAATGGTGGAAAG | ZFN expression |
| ROC 5.2 | GAACGGAACAGGCGGTGAGTC | *ROC1* expression |
| ROC 3.3 | CCACAGGCTTCGTCGGCTTTC | *ROC1* expression |
| SP154 | GGTGGTACTTTTAAGGCAATTCAG | Southern blot probe |
| SP155 | GACAGAATTCCGGTGTTTGTAGAC | Southern blot probe |
| SP156 | GGTGAGTTAGTGGAAGCAGTTGAC | Southern blot probe |
| SP157 | GTCCCATTCAACTATCTTGGTAAG | Southern blot probe |
